# Risk and Protective Factors Associated with Increased Burden in Caring for Children: An Observational Study of Japanese General Households

**DOI:** 10.3390/pediatric17030068

**Published:** 2025-06-18

**Authors:** Tomo Nonoyama

**Affiliations:** Nursing Science, Department of Integrated Health Sciences, Graduate School of Medicine, Nagoya University, Nagoya 461-8673, Aichi, Japan; nonoyama.tomo.s1@f.mail.nagoya-u.ac.jp; Tel.: +81-52-719-1386

**Keywords:** special needs, child care, care burden, infant, Japan

## Abstract

Background/Objectives: Increased burdens on caregivers of infants and toddlers significantly affect caregivers’ quality of life and health. Although adequate care during infancy contributes to child development and special health care needs affect caregiver burden, the risk factors for and protective factors against increased caregiver burden remain unclear. We aimed to evaluate children’s health care needs and required caregiving time and identify factors associated with increased caregiver burden. Methods: We conducted an online survey of 287 Japanese caregivers who were randomly selected from a web panel and were raising children aged <4 years. The survey comprised a sociodemographic data form, Children with Special Health Care Needs (CSHCN) Screener, caregiving time survey form, and questions on increased burden. Needs and caregiving time were evaluated by dividing the participants into CSHCN and non-CSHCN groups. Related factors were analyzed using increased burden as the dependent variable. The chi-square test, Mann–Whitney U test, and modified Poisson regression were used for data analysis. Results: Among the children of the 287 participating caregivers, 16.4% were identified as CSHCN, while 96.9% had no specific diagnosis. Overall, 38.3% of the CSHCN group met only one of the five CSHCN Screener items. The CSHCN group spent significantly more time providing and arranging/coordinating health care. The non-CSHCN group spent significantly more time providing daily care. After adjusting for covariates, increased caregiver burden was significantly associated with a younger age of the child, more caregiving time required 6 months prior to the survey, and providing care for CSHCN. Conclusions: To help reduce the burden of childcare on caregivers of infants and young children, children’s needs should be identified and generous childcare provided from an early age. Early identification of CSHCN and appropriate support for families may help reduce caregiver burden during early childhood.

## 1. Introduction

The burden of childcare has been associated with the onset of depression and other symptoms in caregivers, and it is an important theme in the context of the well-being of children and families [1,2]. In particular, children’s needs, such as their health status, not only have a significant impact on the children themselves in terms of health-related quality of life and mortality, but also have a considerable impact when it comes to the burden of care on the family [3,4]. Additionally, an increased burden on caregivers has a significant negative effect on their quality of life and physical and psychological health [5,6]. Therefore, it is crucial to elucidate the factors associated with increased caregiver burden and explore strategies for its prediction and prevention.

Special health care needs in children are a major factor that affects the caregiver burden associated with childcare [7]. Owing to advances in medical care and technology, the number of children with special health care needs (CSHCN) has increased significantly [8]. CSHCN—defined as “children who have or are at high risk for chronic physical, developmental, behavioral, or emotional disabilities, and who require health-related services of a type or amount beyond those typically needed by children” [9]—have health-service needs related to their daily lives regardless of the type of disease or disability [10]. The relationship between health care needs and care burden has been reported in studies conducted among cohorts with specific conditions, such as children with cancer, autism spectrum disorder, and tracheostomies [11,12,13]. Although the generalizability of the results of these studies is limited, some authors have reported that the characteristics of the child, such as age, are related to the burden of care; for example, the increased mobility of children with tracheostomies increases the burden of care [13,14].

However, most studies have focused only on factors associated with the level of care burden, while the factors affecting changes in care burden remain unclear. Furthermore, very few studies have addressed the health care needs of children without limiting diseases or disabilities and, at present, the relationship between special health care needs and changes in care burden remains unknown. Therefore, it is necessary to conduct research with a focus on health care needs and changes in care burden among general cohorts without limiting diseases or disabilities.

Infants and young children require extensive care and should not be overlooked when providing caregiving support [11,12,15]. During the infant-and-young-child period, families are more likely to face challenges, while significant developmental changes occur in children [16]. Only few reports have focused on the changes in care burden, and the protective factors against an increased care burden remain unclear. A fulfilling relationship with children during this developmental stage (e.g., generous childcare) may contribute to stable child development [17,18,19]. Therefore, research with a focus on the increased burden of care and that explores protective factors in a cohort of infants and toddlers is crucial.

In Japan, there is a particularly pressing need for research on the caregiver burden faced by families raising infants and young children. The authors of previous studies have reported that caregivers in Japan, particularly mothers, are prone to experiencing high levels of psychological stress and social isolation during the early stages of child-rearing. However, public support services that address special health care needs are often contingent upon formal diagnoses [20]. Furthermore, the availability of public childcare support services—such as childcare facilities and home-visit programs—varies significantly by region, leading to disparities in caregiver support systems [21]. Despite these challenges, research on support systems for CSHCN without a formal diagnosis remains limited. Therefore, further research within the Japanese context is essential to clarify children’s care needs and facilitate the development of strategies to support caregivers.

Building on previous findings, the aim of this study was to identify risk and protective factors associated with increased caregiver burden among families with children under 4 years of age. Specifically, we examined children’s health care needs and the time required for caregiving to clarify their association with an increased care burden. The findings are expected to improve the understanding of care needs, facilitate early identification of high-risk families, and inform preventive strategies to alleviate caregiver burden.

## 2. Materials and Methods

### 2.1. Study Design and Participants

Considering that the target cohort in this study comprised caregivers of infants and young children with and without special health care needs, we conducted a cross-sectional study using retrospective data obtained through an online survey, which only required a single response and had a low risk of dropout. Using the online survey, we obtained responses regarding the youngest child from caregivers raising children aged 4 years or younger living in the Tokai-Hokuriku region of Japan.

To maximize participation in this online survey, the questions were simplified and phrased in accessible language. Additionally, at the beginning of the survey, it was clearly stated that anonymity will be ensured and personal information protected. The social significance of the research was also emphasized, and participants were given the flexibility to respond to the survey at any time within a 24 h period.

The survey participants were randomly selected from the web panel of Macromill Inc. (Tokyo, Japan) [22,23], which is an online survey company with a large web panel of pre-registered survey participants and a wide presence in Japan. Macromill Inc. employed stratified random sampling based on region, age, and other demographic variables to reduce sampling bias and ensure representativeness of the target population. The survey was conducted from July to December 2024 and targeted caregivers who met the following conditions based on registered information from the web panel.

### 2.2. Eligibility Criteria

The inclusion criteria were:

- Living in the Tokai-Hokuriku region of Japan with their child;
- Being at least 20 years old;
- Raising a Japanese child aged 4 years or younger;
- Parent or guardian who understands the content of the research and voluntarily agrees to participate.

The exclusion criteria were:

- The child who is the subject of the response has already been diagnosed with a disease or disability;
- The inability to respond to the online survey due to communication issues;
- Incomplete data collection form.

### 2.3. Data Collection

Data were collected using a parent and child sociodemographic data form, CSHCN Screener, caregiving time (6 months prior) survey form, and increase-in-care-burden questionnaire. The CSHCN Screener is a widely used screening tool and has been validated in previous studies; however, internal consistency indices such as the Cronbach α are not typically reported owing to its categorical design. The other items—including caregiving time, perceived increase in care burden, and sociodemographic data—were single or study-specific items and thus not subject to internal consistency assessment.

#### 2.3.1. Parent and Child Sociodemographic Data Form

The parent and child sociodemographic data form was created by the researcher with reference to previous literature [24,25,26,27]. It comprised questions regarding the parents’ age, sex, marital status, and household income, and the child’s age (in months), sex, and frequency of health changes (often, occasional, not much, none). Because the children were infants and toddlers aged 0–4 years, age was recorded in 1-month rather than 1-year units.

#### 2.3.2. CSHCN Screener

The CSHCN Screener was used to identify the details of the children’s health care needs. This gold-standard tool was developed from the research of Bethell et al. [28,29] with the aim of conducting surveys based on clear definitions of and criteria for CSHCN. It was translated into Japanese with permission from The Child & Adolescent Health Measurement Initiative for the purpose of this study. The Japanese version of the CSHCN Screener used in this study was translated in accordance with the guidelines provided by the developers. The translated Japanese version has been used in previous studies conducted in Japan, but formal validation results for Japanese individuals have not yet been published.

This screening tool consisted of the following five items. If the child met any of the five criteria, they were considered a CSHCN. If the caregiver answered “yes” to any of the items and the condition had continued for longer than 1 year or was expected to continue for longer than 1 year, they were considered to meet the criterion.

1. Needs/uses prescription medications;
2. Needs/receives more medical care than usual;
3. Limited ability to do things (functional limitations);
4. Needs/receives occupational, physical, or speech therapy (specialized therapies);
5. Needs/receives emotional, behavioral, or developmental treatment/counseling.

#### 2.3.3. Caregiving Time Survey Form

This form was used to assess the amount of caregiving time, defined as the total number of hours per week that the caregiver spent providing care for the child, as recalled from 6 months prior. Based on studies in which the researchers investigated the amount of time spent caring for CSHCN, the form comprised three sections [25,26]. The types of care included in each section were as follows:

1. Providing health care for the child at home: rehabilitation (including home-visit rehabilitation), therapy other than rehabilitation (including psychological and music therapy by specialists), taking and administering medicine (including eye drops and inhalation), and commuting to the hospital;
2. Arranging or coordinating health care for the child: administrative procedures with other organizations (including correspondence with home-visit facilities and government offices), conferences (including discussions and consultations with professionals regarding childcare and treatment);
3. Providing daily care: meals/breastfeeding, taking baths, and lulling the child to sleep.

Total caregiving time was calculated as the total time spent on the three types of care.

#### 2.3.4. Increase in Care Burden Questionnaire

In this questionnaire, caregivers were required to respond to the question, “Compared to 6 months ago, has the burden of caring for your child increased?” Given that the caregiver’s feelings regarding the burden of care imposed on them are important in supporting them in this context, we used a subjective and straightforward response as the outcome.

According to previous studies [30,31], recall bias increases when the recall period exceeds 6 months; therefore, currently perceived caregiving time and care burden were compared with those of a maximum of 6 months prior. A 6-month comparison period was selected to balance the need to capture meaningful changes in caregiving burden with minimizing recall bias. This timeframe is also consistent with that of previous studies on caregiver stress and health service utilization [32,33].

### 2.4. Ethical Considerations

The study protocol was approved by the Bioethics Review Committee of the Nagoya University Graduate School of Medicine (approval no. 2024-0136-2). For caregivers who met the criteria for participation, the research explanation document and consent form were presented on the website, and the survey was administered to those who provided consent via the online consent form. The research explanation document and consent form indicated that participation was voluntary, consent could be withdrawn at any time, personal information would not be disclosed to comply with the “principle of confidentiality,” and data would be stored strictly.

### 2.5. Statistical Analysis

The data are presented as numbers, percentages, medians, and interquartile ranges, as appropriate. The Shapiro–Wilk test was used to assess data normality, and nonparametric tests were applied to non-normally distributed variables.

The frequencies and percentages of the sociodemographic characteristics are shown for the total sample, CSHCN group, and non-CSHCN group. Medians were calculated for the age of the parents and children, and the distributions were compared using the Mann–Whitney U test; other variables were compared using the chi-square test.

To assess the children’s health care needs and required caregiving time, the number of applicable CSHCN Screener items and corresponding child counts were tabulated. Caregiving time (health care provision, coordination, daily care, and total) was summarized using medians, interquartile ranges, and minimum/maximum values, and compared between groups using the Mann–Whitney U test.

To explore factors associated with an increase in caregiver burden, participants were classified into two groups based on their response (yes/no) to the question, “Compared to 6 months ago, has the burden of caring for your child increased?”

We conducted a modified Poisson regression analysis to examine factors associated with increased caregiver burden. First, univariate analyses were conducted for each variable. Subsequently, two-step multivariate analyses were performed: in step one, adjustments were made for child sex and age; and in step two, for sex, age, diagnosis, health change frequency, total caregiving time, and CSHCN status. Adjusted risk ratios (RRs) and 95% confidence intervals (CIs) were calculated. Covariates were selected based on their clinical relevance and prior studies [11,12,15].

All analyses were performed using IBM SPSS Statistics (version 29.0; IBM Corp., Armonk, NY, USA). Statistical significance was set at *p* < 0.05.

## 3. Results

Responses were obtained from 287 participants that were selected at random from the web panel. Table 1 shows the distribution of the sociodemographic data of the caregivers and children, as well as the children’s special health care needs and required amount of caregiving time.

Of the 287 children, 47 (16.4%) were CSHCN. In terms of the parents’ attributes, the most common age group was the 30s (62.4%), with a median age of 34 years and a range of 22 years to 64 years. Most of the parents (97.2%) were married. The most common household income bracket was middle class (39.7%).

In terms of the children’s attributes, the sex breakdown was 53.3% male and 46.7% female. The median age was 23 months, with a range of 6 months to 48 months. The majority (99.2% and 85.1%) of the children of caregivers in the non-CSHCN and CSHCN groups, respectively, had not been diagnosed with a specific disease or disorder. Specific diagnoses among those who had been diagnosed included congenital heart disease, Down syndrome, and food allergies. In terms of the frequency of changes in physical condition, in the non-CSHCN group, the total of “none” and “not much” accounted for over 60% (64.2%), while in the CSHCN group, the total of “often” and “occasional” accounted for over 60% (63.9%). A comparison between the CSHCN and non-CSHCN groups revealed significant differences in diagnosis and frequency of health changes.

Regarding the distribution of special health care needs in the CSHCN group, 38.3% of the respondents selected only one of the five CSHCN Screener items. When the numbers of respondents who selected one and two items were combined, more than half (57.4%) selected at least one item. In terms of the content of special health care needs, the need for/use of prescription medicines was the most common (66.0%), followed by the above-average need for/use of services (48.9%).

In terms of caregiving time, the CSHCN group tended to require relatively more time for “providing health care” and “arranging or coordinating health care”; however, while the minimum and maximum values for the CSHCN group were 0.0–70.0 and 0.0–8.0, respectively, and the minimum and maximum values for the non-CSHCN group were 0.0–30.5 and 0.0–8.0, respectively, there were also households in the non-CSHCN group in which the children required a certain amount of caregiving time. The median value for “providing daily care” in the non-CSHCN group was relatively high. The results of the Mann–Whitney U test also revealed that the caregiving time required in the CSHCN group was significantly longer for “providing health care” and “arranging or coordinating health care”, while the time required in the non-CSHCN group was significantly longer for “providing daily care,” with no significant difference in total time.

Table 2 shows the results of the univariate analysis of the association between each variable and an increased care burden compared to 6 months previously, using each variable as an independent variable; the results of the modified Poisson regression with sex and age as covariates; and the results of the modified Poisson regression with sex, age, diagnosis, frequency of health changes, and whether the children met the criteria for CSHCN as covariates.

Even after adjusting for various covariates, the child’s age (RR = 1.02, 95% CI = 1.00–1.04), total caregiving time (hours) 6 months prior (RR = 0.97, 95% CI = 0.95–0.99), and whether the child met the criteria for CSHCN (RR = 2.19, 95% CI = 1.19–4.05) were found to be significantly associated with an increased care burden compared to 6 months prior. Specifically, the child’s age and whether they met the criteria for CSHCN were positively correlated with, and considered risk factors for, increased care burden. However, total caregiving time (hours) 6 months prior showed a negative correlation, suggesting that the more time spent on care 6 months prior, the lower the risk of increased care burden.

## 4. Discussion

In this study, a cohort of infants from general households in Japan was used to identify children’s health care needs and required caregiving time. We found that an increase in caregiver burden was related to the child’s age, total required caregiving time 6 months prior, and whether the child met the criteria for CSHCN. To the best of our knowledge, this is the first study to identify factors related to an increase in caregiver burden in a cohort of infants from general households.

The characteristics of the cohort used in this study were average and not biased, with parents’ ages and household incomes being average. The sociodemographic data were similar to those of a previous cohort survey conducted by a Japanese government agency [34], and the data in this study can be considered representative to a certain extent. After confirming this premise, we identified the actual health care needs of the children.

The prevalence of CSHCN in this cohort of infants and toddlers was 16.4%. In the United States, the prevalence of CSHCN among children younger than 18 years was reported to be 12.8% (approximately 9.4 million) in 2001, and 19% (approximately 14 million) in 2022 [21,35]. In a Japanese study conducted between 2012 and 2015, the prevalence of CSHCN among children with an average age of 9.74 years was 12.5% [36]. The proportion of CSHCN in this study was consistent with that reported previously. As mentioned above, infants and young children constitute a population associated with a particularly high care burden. Nevertheless, only few researchers have investigated the actual state of special health care needs in infants and young children, and data from cohorts outside the United States are limited [37]. Our results clarified that infants and young children have special health care needs similar to those of other children.

Furthermore, although 85.1% of the CSHCN had not been diagnosed with a specific disease or disorder, 63.9% had experienced occasional or frequent changes in their health. The authors of previous studies have cited intellectual disability and emotional disorders as examples of diagnoses received by CSHCN [7]. The target population in this study was infants and young children aged 0–4 years. Therefore, although not clearly identified, it is possible that the study included children who were likely to receive such a diagnosis in the future. However, past studies have shown that 30–50% of CSHCN experience occasional or frequent health changes [38]. Herein, the frequency of changes in physical condition was higher than that reported in previous studies, indicating the significant needs of CSHCN in this cohort. Having special health care needs without having been clinically diagnosed may be a characteristic of infants and young children. Therefore, in such a cohort, it should be considered that caregivers may face high health care demands from their children, regardless of whether the child has received a diagnosis.

Most of the needs of CSHCN were related to one or two of the five CSHCN Screener items. Special health care needs were often related to the need for/use of prescription medicines or the above-average need for/use of services. The number of CSHCN Screener items that apply to the child indicates the complexity of their requirements [39]. Therefore, the health care needs of the CSHCN in this cohort were not extremely complex. However, the need for/use of prescription medicines and above-average need for/use of services indicate the possibility of dependence on some kind of medical and welfare service, even without a diagnosis, rendering early intervention desirable [21]. Special health care needs can change depending on the living environment and family involvement. Furthermore, an increase in these needs is reportedly more common in children aged 6 years and older [40]. Therefore, the results of the present study suggest that, to prevent an increase in children’s care needs, it is important to identify such needs from infancy and provide early support to the family.

Herein, caregivers spent approximately 16 h per week caring for their children, and regardless of whether the child met the criteria for CSHCN, a large proportion of the time was spend providing daily care. However, while there was no significant difference in the total caregiving time, the time spent providing and arranging or coordinating health care was significantly greater in the CSHCN group, whereas the time spent providing daily care was significantly greater in the non-CSHCN group. Studies on caregivers of children have revealed that caregivers of CSHCN experience a greater burden, particularly in terms of coordinating care and managing schedules [7,41,42]. However, for infants and young children, the time spent providing childcare, such as feeding and lulling them to sleep, is considered essential and cannot be limited. These results suggest that in some households with CSHCN, caregivers may spend more time providing health care and other services in addition to daily care, and they may be bearing a greater burden within the limited time they have available to provide childcare. To meet infants’ needs and contribute to their stable development, caregivers should be supported to ensure that they have sufficient time to spend on daily care. This requires smooth health care provision and care coordination guided by a detailed understanding of the needs of each child in each family.

Herein, the factors associated with increased care burden included the child’s age, total caregiving time (hours) 6 months prior and whether the child met the criteria for CSHCN. Thus, in this cohort, a child’s older age and having special health care needs were risk factors for an increase in care burden compared to 6 months prior, whereas investing more caregiving hours 6 months prior was a protective factor against an increase in care burden.

Older children reportedly place a greater care burden and have increased needs [14,40]. Our results also showed that, in infants and young children, the burden of care tended to increase as the child got older. Therefore, to prevent an increase in the burden of care, avoid a decline in the quality of life of caregivers, and prevent the onset of mental disorders, it is important to intervene at an earlier stage.

Moreover, when providing support to families with CSHCN, clear identification of those with a high risk for an increased caregiving burden is useful for effective screening. It can be assumed that the baseline care burden is relatively high when a child has chronic health care needs [43]. However, our results revealed that, regardless of whether an infant has been diagnosed with a specific disease or disability, if the child had high health care needs, the burden was likely to increase in a short period. Therefore, care providers should identify high-risk households, regularly check the care burden, and build a support system.

Our results also suggest that it is important to provide more intensive care from an early stage to prevent an increase in care burden in the infant cohort. This result aligns with the finding that prolonged intensive care during infancy contributes to stable child development [18]. Our finding that past caregiving time may function as a protective factor could reflect caregivers’ adaptation over time as well as the availability of external support systems, such as family, community resources, and professional support. Additionally, it may be attributed to the study population, and may only be applicable to cohorts of relatively young children. Further research is needed to elucidate these potential mechanisms. From a public health perspective, these findings emphasize the importance of early intervention programs that support caregivers through education, home visits, and accessible pediatric medical care. For example, parenting support programs, respite care services, and early screening for developmental concerns may reduce caregiver burden and promote healthy child development.

To prevent the subjective care burden of caregivers from increasing owing to the provision of support, it is important to identify the needs of each child and the care that requires time, and that the support provided by professionals is neither excessive nor insufficient. For example, in cases where parents have difficulties with their child’s feeding or nighttime crying, it may be possible to reduce the burden on the parents without interrupting adequate childcare through continuous support from midwives or temporary respite care. When parents struggle with weaning their child, guidance from speech–language pathologists or dietitians may facilitate the child’s stable development while also allowing the family to spend more time together to enjoy meals.

The results of this study suggest that reducing the care burden on caregivers of infants and young children requires specialized, tailored support that meets the needs of each child. Additionally, it is important to ensure that caregivers have sufficient time for care and can provide generous care from an early stage.

This study has several notable strengths. First, targeting a cohort of infants and toddlers from ordinary households facilitated a discussion of the care burden on caregivers of infants and toddlers and support to reduce that burden. Second, investigating the factors associated with changes in care burden allowed for an analysis focused on the increase in this burden and the provision of recommendations for support.

### 4.1. Limitations

This study has certain notable limitations. First, the target cohort consisted of infants and young children from general Japanese households, and responses were obtained through an online survey. Therefore, there is a possibility of variation in responses. In addition, as the study was conducted in a specific region of Japan, the generalizability of the findings may be limited. A potential limitation of this study is the cultural and linguistic specificity of the response options regarding the frequency of health changes. The original Japanese questionnaire was carefully designed to capture subtle differences in health experiences using expressions that are commonly distinguished in Japanese daily life. However, these nuanced categories may not directly translate into English or other languages without potential loss or distortion of meaning. In the future, researchers should accumulate and compare studies on CSHCN in diverse cohorts with different cultural or physical backgrounds. Second, regarding caregiving time, responses were obtained based on the content of care that is generally considered with reference to previous studies. It is difficult to cover all aspects of infant care, and there may be other potential factors apart from the measured caregiving time. However, no evidence suggests that these potential factors had a significant impact on the analysis. It is important to verify the results of this study by conducting large-scale or longitudinal surveys.

### 4.2. Further Research Directions

The findings of this study offer valuable insights for various stakeholders, including primary care providers of children, public health policymakers, and families raising infants and toddlers. These groups can utilize the results to promote the early identification of caregiving challenges, encourage family-centered care, and more effectively leverage available social and community resources for parenting support.

Future research should be aimed at validating these findings in larger and more diverse populations to enhance generalizability. Studies targeting children in different age groups or with varying degrees of health care needs are also warranted. Moreover, research evaluating the impact of specific interventions or policies—such as caregiver education, early screening programs, or integrated support services—would be beneficial to translate these insights into practical strategies that help prevent caregiver burden and foster healthy child development.

## 5. Conclusions

We evaluated the health care needs and caregiving time required by infants in a sample of general households from the Tokai-Hokuriku region in Japan that were recruited via an online panel. We found that the increase in care burden compared to 6 months prior was associated with the child’s age, the caregiving time invested 6 months prior, and whether the child met the criteria for CSHCN. The same rate of CSHCN (16.4%) was found among infants in general households as that found in previous studies. To translate these findings into practice, regular use of CSHCN screeners in primary care settings will enable the early identification of families at risk of increased caregiving burdens. Furthermore, strengthening integrated family support systems—including coordination between health, welfare, and childcare services—will prevent caregivers from facing these challenges alone and help sustain a nurturing environment that supports healthy child development. When supporting the caregivers of infants, it is important to recognize that they may encounter substantial health care demands from their children, regardless of whether the child has received a formal diagnosis. It is also important to establish a support system that includes the family, such that caregivers do not face an increase in their burden while maintaining a nurturing environment that contributes to the stable development of the child.

## Figures and Tables

**Table 1:** Participant characteristics.

Characteristics	Total	CSHCN	Non-CSHCN	
	n (%)/Median [IQR]			*p*-Value
n (%)	287 (100.0)	47 (16.4)	240 (83.6)	
Caregiver characteristics				
Sex				
Male	90 (31.4)	13 (27.7)	77 (32.1)	0.550
Female	197 (68.6)	34 (72.3)	163 (67.9)	
Age (years)				
20–29	55 (19.2)	13 (27.7)	42 (17.5)	0.173
30–39	179 (62.4)	24 (51.1)	155 (64.6)	
≥40	53 (18.5)	10 (21.3)	43 (17.9)	
Median (IQR)	34.0 [31.0–38.0]	34.0 [29.0–39.0]	34.0 [31.0–38.0]	0.654
Marital status				
Married	279 (97.2)	47 (100.0)	232 (96.7)	0.204
Unmarried	8 (2.8)	0 (0.0)	8 (3.3)	
Annual income of parents (JPY)				
2,000,000–3,999,999 (low)	32 (11.1)	6 (12.8)	26 (10.8)	0.834
4,000,000–7,999,999 (middle)	114 (39.7)	16 (34.0)	98 (40.8)	
≥8,000,000 (high)	60 (20.9)	10 (21.3)	50 (20.8)	
No answer	81 (28.2)	15 (31.9)	66 (27.5)	
Child characteristics				
Sex				
Male	153 (53.3)	27 (57.4)	126 (52.5)	0.534
Female	134 (46.7)	20 (42.6)	114 (47.5)	
Age, months (years)				
6–11 (0)	76 (26.5)	15 (31.9)	61 (25.4)	0.359
12–23 (1)	71 (24.7)	7 (14.9)	64 (26.7)	
24–35 (2)	79 (27.5)	15 (31.9)	64 (26.7)	
36–48 (3–4)	61 (21.3)	10 (21.3)	51 (21.3)	
Median (IQR)	23.0 [11.0–34.0]	24.0 [8.0–33.0]	23.0 [11.0–34.8]	0.537
Diagnosis				
Diagnosed	19 (3.1)	7 (14.9)	2 (0.8)	<0.001 **
Non-diagnosed	278 (96.9)	40 (85.1)	238 (99.2)	
Frequency of health changes				
Often	28 (9.8)	10 (21.3)	18 (7.5)	<0.001 **
Occasional	88 (30.7)	20 (42.6)	68 (28.3)	
Not much	79 (27.5)	13 (27.7)	66 (27.5)	
None	92 (32.1)	4 (8.5)	88 (36.7)	
Total number of applicable items in the CSHCN Screener				
1		18 (38.3)		
2		9 (19.1)		
3		9 (19.1)		
4		3 (6.4)		
5		8 (17.0)		
Numbers for each item applicable				
Needs/uses prescription medicines		31 (66.0)		
Above-average need for/use of services		23 (48.9)		
Functional limitations		21 (44.7)		
Needs/receives specialized therapies		19 (40.4)		
Needs/receives emotional, behavioral, or developmental treatment/counseling		21 (44.7)		
Caregiving time (6 months prior)				
Providing health care	0.0 [0.0–1.0]	1.0 [0.0–4.0]	0.0 [0.0–0.0]	<0.001 **
Arranging or coordinating health care	0.0 [0.0–0.0]	0.0 [0.0–1.0]	0.0 [0.0–0.0]	<0.001 **
Providing daily care	15.0 [9.0–22.0]	11.5 [7.0–17.0]	16.0 [9.6–22.9]	0.011 *
Total hours	16.0 [10.0–24.0]	13.2 [10.5–28.5]	17.0 [10.0–24.0]	0.561

Data are presented as medians [IQR] or numbers (%), as appropriate. Values are expressed as number (%) for categorical variables and median [interquartile range] for continuous variables. Abbreviations: CSHCN, children with special health care needs; IQR, interquartile range. The chi-square test was used to compare the frequencies of all categorical variables. The Mann–Whitney U test was used to compare the distribution of caregivers’ age, children’s age, and caregiving time. * *p* < 0.05, ** *p* < 0.001. Statistically significant findings are indicated in bold.

**Table 2:** Association of each variable with subjective increases in care burden.

Characteristics	Care Burden Increase *n* = 65	No Care Burden Increase *n* = 222	RR (95% CI)	*p*-Value	Adjusted RR (95% CI) ^a^	Adjusted *p*-Value ^a^	Adjusted RR (95% CI) ^b^	Adjusted *p*-Value ^b^
	*n* (%)/Mean (SD)							
Sex								
Male	32 (49.2)	121 (54.5)	ref					
Female	33 (50.8)	101 (45.5)	1.18 (0.72–1.92)	0.510	1.16 (0.71–1.89)	0.551	1.18 (0.72–1.93)	0.516
Age (months)	27.4 (12.5)	21.8 (12.9)	1.03 (1.01–1.05)	0.008 *	1.03 (1.01–1.05)	0.008 *	1.02 (1.00–1.04)	0.019 *
Diagnosis								
Non-diagnosed	61 (93.8)	217 (97.7)	ref					
Diagnosed	4 (6.2)	5 (2.3)	2.03 (0.74–5.57)	0.171	1.64 (0.59–4.57)	0.343	1.05 (0.35–3.13)	0.936
Frequency of health changes								
None	23 (35.4)	69 (31.1)	ref					
Not much	17 (26.2)	62 (27.9)	0.86 (0.46–1.61)	0.639	0.86 (0.46–1.62)	0.645	0.84 (0.44–1.60)	0.590
Occasional	18 (27.7)	70 (31.5)	0.82 (0.44–1.52)	0.524	0.78 (0.42–1.46)	0.440	0.74 (0.39–1.41)	0.361
Often	7 (10.8)	21 (9.5)	1.00 (0.43–2.33)	1.000	0.88 (0.38–2.07)	0.774	0.80 (0.32–1.98)	0.626
Total caregiving time (hours)	15.3 (11.9)	22.5 (16.3)	0.97 (0.94–0.99)	0.005 *	0.97 (0.94–0.99)	0.010 *	0.97 (0.95–0.99)	0.014 *
Child meets criteria for CSHCN								
No	47 (72.3)	193 (86.9)	ref					
Yes	18 (27.7)	29 (13.1)	1.96 (1.14–3.37)	0.016 *	2.01 (1.17–3.47)	0.012 *	2.19 (1.19–4.05)	0.012 *

Participants were categorized based on their responses to the question “Compared to 6 months ago, has the burden of caring for your child increased?” This grouping was not used in Table 1. Abbreviations: CSHCN, children with special health care needs; CI, confidence interval; RR, risk ratio; ref, reference. For the risk ratio, the value per month is shown for age and the value per hour is shown for total hours. *p*-values were calculated using modified Poisson regression analysis adjusted for an increase in care burden. ^a^ *p*-values were calculated using modified Poisson regression analysis, including sex and age as factors and covariates. ^b^ *p*-values were calculated using modified Poisson regression analysis, including sex, age, diagnosis, frequency of health changes, total caregiving time (hours), and whether the child met the criteria for CSHCN as factors and covariates. * *p* < 0.05. Statistically significant findings are indicated in bold.

## Data Availability

The data presented in this study are available on request from the corresponding author. (The data are not publicly available due to privacy.)

## Abbreviations

The following abbreviations are used in this manuscript:

CI	Confidence interval
CSHCN	Children with special health care needs
RR	Relative risk
